# Draft Genome Sequence of a Multidrug-Resistant Sequence Type 231 Outbreak-Associated Clone of *Klebsiella pneumoniae*, KP41-2015, Producing OXA-232 Carbapenemase

**DOI:** 10.1128/genomeA.00604-17

**Published:** 2017-07-06

**Authors:** M. H. F. Abdul Momin, A. Liakopoulos, D. W. Wareham

**Affiliations:** a Antimicrobial Research Group, Blizard Institute, Barts & the London School of Medicine and Dentistry, Queen Mary University of London, London, United Kingdom; b Microbiology Laboratory Services, RIPAS Hospital, Bandar Seri Begawan, Brunei; c Department of Bacteriology and Epidemiology, CVI of Wageningen University, Lelystad, The Netherlands; d Division of Infection, Barts Healthcare NHS Trust, London, United Kingdom

## Abstract

Carbapenem-resistant *Klebsiella pneumoniae* infection is a rising public health threat due to limited therapeutic options. Here, we report the genome sequence of a multidrug-resistant *K. pneumoniae* sequence type 231 (ST231) strain associated with an outbreak of infections in an intensive care unit that carries a unique complement of resistance determinants.

## GENOME ANNOUNCEMENT

*Klebsiella pneumoniae* is an opportunistic pathogen of human mucosal surfaces resposible for community- and hospital-acquired bloodstream, respiratory, intra-abdominal, and urinary tract infections (1). Carbapenem-resistant *Klebsiella pneumoniae* (CRKP) infections have been particularly associated with high mortality in infected patients (2).

*K. pneumoniae* KP41-2015 was isolated in 2015 from a wound swab during an outbreak of CRKP infections in patients hospitalized in two intensive care units in Brunei Darussalam (3). The strain belonged to the sequence type 231 (ST231) lineage and exhibited resistance to aminoglycosides, cephalosporins, carbapenems, fluoroquinolones, and sulfonamides but remained susceptible to polymyxins and ceftazidime-avibactam.

Genomic DNA from KP41-2015 was subjected to whole-genome sequencing using 2 × 250-bp paired-end reads on an Illumina MiSeq platform (Illumina, Inc., San Diego, CA), generating a total of 338,242 reads with an average length of 521 bp. The generated reads were trimmed using the Trimmomatic algorithm (version 0.36) (4) and their quality assessed by in-house scripts combined with SAMtools (version 1.3.1) (5), BedTools (version 2.25.0) (6), and BWA-mem (version 2) (7) algorithms. High-quality filtered reads were subsequently assembled *de novo* using SPAdes algorithm (version 3.7.1) (8) into 98 scaffolds, with a minimum length of 211 bp and an *N*_50_ of 316,659 bp. The sequence coverage of the *de novo* assemblies was approximately 190 reads per assembled base. The draft genome sequence of KP41-2015 revealed a genome size of 5,692,661 bp, with an average G+C content of 56.94%.

Provisional annotation using the *ab initio* gene finder algorithm Prokka (version 1.11) (9) revealed a total of 5,570 coding sequences (CDSs), including at least 82 tRNAs, 23 rRNAs (8 complete and 15 partial), and 19 ncRNAs. The determination of its antimicrobial resistome using ResFinder (10) revealed a unique complement of genes conferring resistance to aminoglycosides [*aph(3′)-Ic*, *aacA4*, and *rmtf*], β-lactams (*bla*_SHV-11_, *bla*_TEM-1b_, *bla*_CTX-M-15_, and *bla*_OXA-232_), fluoroquinolones (*oqxA*, *oqxB*, and *qnrS1*), fosfomycin (*fosA*), macrolides, lincosamides and streptogramin B [*erm*(42)], phenicols (*catA1* and *floR*), rifampin (*arr-2*), and sulfonamides (*sul2*). PlasmidFinder (11) confirmed the presence of the A/C_2_, ColK(P_3)_, FIB, and FII_(K)_ replicon types, as we have shown earlier based on PCR-based replicon typing (Diatheva, Fano, Italy), while PHAST (12) revealed the presence of four intact, one incomplete, and two putative prophages. *K. pneumoniae* KP41-2015 contains genes for iron acquisition, such as *kfuA* and *kfuC* and several virulence genes including *fyuA*, *irp1*, *irp2 ybtA*, *ybtE*, *ybtP*, *ybtQ*, *ybtS*, *ybtT*, *ybtU* and *ybtX* (yersiniabactins), as well as *mrkD* and *mrkH* (type 3 fimbriae formation).

This is the first genome sequence, to our knowledge, describing the genetic factors that have contributed to the recent emergence of this multidrug-resistant clone that appears to be disseminating throughout Southeast Asia.

### Accession number(s).

The draft genome sequence of *K. pneumoniae* KP41-2015 has been deposited at the DDBJ/EMBL/GenBank databases under the accession number MCNI00000000. The version described in this paper is the first version, MCNI01000000.
